# 

**Published:** 1891-06-27

**Authors:** 


PRICE ONE PENNY.

No. 248, Vol. X.—June 27th, 1891.

[Registered at the General Post Office as a Newspaper for transmission in the United Kingdom and Abroad.]

WITH EXTRA NURSING SUPPLEMENT.
Per Annum, 6s. 6d.; post free.
Monthly Parts, 6d.; post free 8d.
Ditto per Annum, 8s., post free.

### THE HOSPITAL

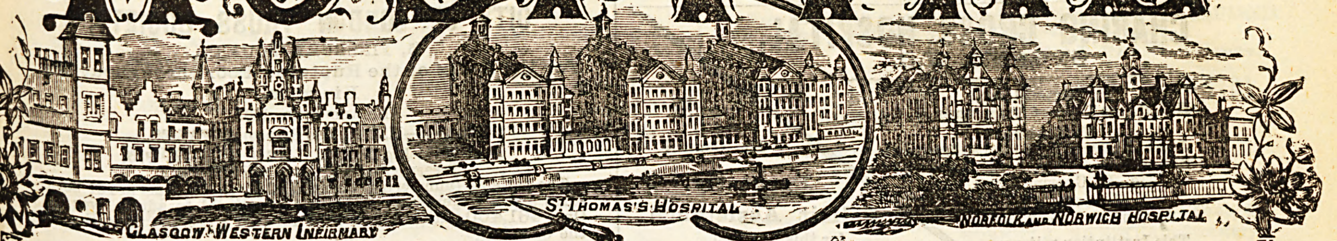

A WEEKLY JOURNAL OF
Science, Medicine, Nursing and Philanthropy.

SATURDAY, JUNE 27th, 1891.

**Articles of Scientific Faith** . . . . . . . 145
**Passing Topics.**—The Programme of the Saturday Fund . 146
**Recreation in Relation to the Health of the People**—IV.—Recreation for the Mind . . . . . . . 147
**The History and Capabilities of Herbal Simples.** By W. T. Fernie, M.D.—XXXIII.—The Elder (*continued*)—Elecampane . . . . . . . . . . 148
**Everybody's Page** . . . . . . . . . 149
**Notes and News** . . . . . . . . . 150
**General Charities** . . . . . . . . . 151
**Annotations.**—The Sunday Collections—The General Practitioners' Union—Clerical Specialism . . . . . 152
**Scraps and Gleanings** . . . . . . . . 151
**The Practitioners' Mirror and Retrospect**—
Medicine.—Influenza, 153; Varix of the Tongue . . 154
Therapeutics.—Styrone . . . . . . . 154
Rhinology.—Deviations of the Nasal Septum . . . 154
New Drugs, Appliances, and Things Medical.—Sanitas Disinfector—Malto Carnis (Caffyn)—The Zona Patent Elastic Occasional Belt for Ladies . . . . . . 155
**Books, &c., Received** . . . . . . . . 155
**The Infinitely Small** . . . . . . . . 156
**The Student of Medicine.**—Editorial Notes—Appointments—Vacancies—Scholarships and Prizes—Pass Lists.

EXTRA SUPPLEMENT—THE NURSING MIRROR.

**En Passant.**—Sick Nursing at Stalybridge—Benefit Association—Richmond House—Northampton Nurses—The New Departure at Queen's . . . . . . . . . lxxi

**The Lords' Committee** . . . . . . . . lxxii

**Asylum Articles.** I.—The Building . . . . . lxxii

**Nursing Medals and Certificates.**—St. John the Divine lxxiii

**Appointments** . . . . . . . . . . lxxiii

**For Reading to the Sick**—The Other Side . . . . lxxiii

**Notes from Australia** . . . . . . . . lxxiv

**Notes and Queries** . . . . . . . . . lxxiv

**Everybody's Opinion.**—The Dulness of Domestic Service—Boston Infirmary—District Nursing . . . . . lxxv

**Presentations—Montrose Asylum** . . . . . lxxv

**Off Duty.**—The Message of the Roses . . . . . lxxvi

**To the Fore** . . . . . . . . . . lxxvi

**Amusements and Relaxation** . . . . . . lxxvi

